# A Portable Smartphone-Based 3D-Printed Biosensing Platform for Kidney Function Biomarker Quantification

**DOI:** 10.3390/bios15030192

**Published:** 2025-03-18

**Authors:** Sangeeta Palekar, Sharayu Kalambe, Jayu Kalambe, Madhusudan B. Kulkarni, Manish Bhaiyya

**Affiliations:** 1 Department of Electronics Engineering, Ramdeobaba University, Nagpur 440013, MH, India; 2 Department of Computer Science Engineering, Symbiosis Institute of Technology, Nagpur 440008, MH, India; 3 Department of Electronics and Communication Engineering, Manipal Institute of Technology, Manipal Academy of Higher Education (MAHE), Manipal 576104, KA, India

**Keywords:** point-of-care-testing, diagnostics, health monitoring, kidney biomarker, colorimetric, 3D printing, smartphone

## Abstract

Detecting kidney function biomarkers is critical for the early diagnosis of kidney diseases and monitoring treatment efficacy. In this work, a portable, 3D-printed colorimetric sensor platform was developed to detect key kidney biomarkers: uric acid, creatinine, and albumin. The platform features a 3D-printed enclosure with integrated diffused LED lighting to ensure a controlled environment for image acquisition. A disposable 3D-printed flow cell holds samples, ensuring precision and minimizing contamination. The sensor relies on colorimetric analysis, where a reagent reacts with blood serum to produce a color shift proportional to the biomarker concentration. Using a smartphone, the color change is captured, and RGB values are normalized to calculate concentrations based on the Beer-Lambert Law. The system adapts to variations in smartphones, reagent brands, and lighting conditions through an adaptive calibration algorithm, ensuring flexibility and accuracy. The sensor demonstrated good linear detection ranges for uric acid (1–30 mg/dL), creatinine (0.1–20 mg/dL), and albumin (0.1–8 g/dL), with detection limits of 1.15 mg/dL, 0.15 mg/dL, and 0.11 g/dL, respectively. These results correlated well with commercial biochemistry analyzers. Additionally, an Android application was developed to handle image processing and database management, providing a user-friendly interface for real-time blood analysis. This portable, cost-effective platform shows significant potential for point-of-care diagnostics and remote health monitoring.

## 1. Introduction

The prevalence of kidney disease has significantly increased twofold globally during the past decades. Monitoring kidney function is critical for maintaining overall health and well-being, as the kidneys play a vital role in filtering waste products and excess fluids from the blood, regulating electrolytes, and maintaining the body’s acid-base balance [1]. The kidneys are also responsible for producing hormones that regulate blood pressure and control the production of red blood cells. Given these essential functions, any impairment in kidney performance can lead to significant health issues, including chronic kidney disease (CKD), which is a progressive condition that can ultimately lead to kidney failure. Chronic kidney disease often progresses silently, with symptoms becoming apparent only in the later stages [2]. As a result, early detection and regular monitoring are crucial for managing the disease, slowing its progression, and preventing future complications.

Monitoring uric acid, creatinine, and albumin levels is essential for assessing kidney function and diagnosing kidney-related disorders. Uric acid is a byproduct of purine metabolism that the kidneys filter from the blood. Elevated uric acid levels may indicate reduced kidney function, potentially leading to gout and conditions like chronic kidney disease (CKD) or kidney stones. Creatinine, another crucial biomarker, is a waste product from muscle metabolism. It is primarily excreted by the kidneys, making it a key indicator of kidney function. High creatinine levels in the blood suggest that the kidneys are not efficiently filtering waste, which is a hallmark of kidney dysfunction [3,4]. Albumin, a protein normally retained in the bloodstream, can appear in urine if the kidneys are damaged, particularly in cases of diabetic nephropathy. Its elevated levels is an early indicator of kidney damage and is critical for diagnosing and managing kidney disease. Together, these biomarkers provide a comprehensive picture of kidney health, enabling early detection of kidney dysfunction, monitoring of disease progression, and assessment of the effectiveness of therapeutic interventions.

Traditional methods of testing these biomarkers are typically performed in clinical laboratories using sophisticated equipment [5]. The biochemistry analyzer is medical equipment that is frequently used in clinical labs for sensing blood serum. Healthcare professionals and medical practitioners frequently employ biochemistry analysers in a variety of medical settings and laboratories to diagnose, treat, and detect serious conditions, such as health disorders. The biochemistry analyzer works on the principle of Beer-Lambert law and requires complex optical assembly [6]. 

In recent years much research has been done for biochemical blood sensing using methods like electrochemical [7,8], Fluorescence [9], Optical detection [10,11], Colorimetric [12,13,14], and Smartphone-based analyzer [15,16,17]. The biochemistry analyzer is the gold standard used in pathological laboratories and is based on the colorimetric detection principle. The detection mechanism relies on colorimetric biochemical reactions, where the intensity of the developed color correlates with the concentration of the target analyte in the blood serum. Centrifuged serum, which is transparent, is mixed with a specific reagent that induces a measurable color change proportional to the analyte concentration [18,19]. However, this approach has limitations, particularly in resource-limited settings where access to such facilities may be restricted. The need for accessible, accurate, and convenient methods of kidney function monitoring is becoming increasingly crucial, particularly given the rising prevalence of kidney disease worldwide. Portable, user-friendly devices that allow for point-of-care testing (PoCT) offer a promising solution to this challenge. By enabling individuals to monitor their kidney function at home or in remote areas, these devices can facilitate early detection, improve disease management, and ultimately reduce the burden of kidney disease on healthcare systems.

Hence, this work proposes to develop an platform for the blood biomarker sensing capable of overcoming all the mentioned drawbacks. A smartphone-based platform was developed with colorimetric sensing principle based on color image acquisition and analysis. The red (R), green (G), and blue (B) values were extracted from the captured images, and an adaptive calibration algorithm was developed to estimate the concentration of targeted biomarkers. The use of smartphone, image processing techniques and adaptive calibration algorithm subsides the requirement for wavelength filters and minimizes the hardware of the existing analyzers [20]. As a case study, the detailed process for uric acid sensing is explained in this work and the experimental results of the remaining parameters like creatinine, and albumin levels are summarized in this paper. These parameters are required for monitoring Kidney related diseases. All other blood biomarkers working on the colorimetric principle can also be detected with the same platform by utilizing the adaptive calibration for other diseases.

## 2. Material and Methods

### 2.1. Chemicals and Material Used

A uric acid reagent was used for sensing uric acid concentration in blood. The uric acid reagent consists of Phosphate Buffer (85 mmol/L), Uricase (1.6KU), Peroxidase (1.75KU), 4-Amino Antipyrine (0.55 mmol/L), Chromogen, Surfactant, and stabilizers. The uric acid standard solution is prepared in a phosphate buffer to maintain pH stability preventing deviations due to pH fluctuations. The uric acid catalyzed by uricase oxidizes, producing allantoin, carbon di-oxide, and hydrogen peroxide. The resulting hydrogen peroxide then reacts with 4-aminophenazone (4AAP) and DHBS in the presence of peroxidase, forming a colored compound, Quinoneimine. For creatinine, sensing modified Jaffe’s method is used, and the reagent consists of picric acid (20 mmol/L) and sodium hydroxide (15 mmol/L). The creatinine standard solution is dissolved in an alkaline buffer containing sodium hydroxide. For albumin, the reagent consists of succinate buffer (90 mmol/L) and bromocresol green (0.26 mmol/L). The albumin standard is prepared in a succinate buffer to maintain the appropriate pH for binding with Bromocresol Green (BCG). All the chemical reagents were purchased from Icon Enterprises, Nagpur, India. DI water required for standard dilution was procured from Sigma Aldrich, Nagpur, India. The 3D-printed enclosure and flow cell were fabricated using black and white polylactic acid (PLA) filament, respectively. Smartphones from Samsung, OnePlus, and Motorola were utilized for image sensing and color analysis to quantitatively estimate the targeted biomarker. A 12 V-diffused LED strip, purchased from Amazon, was employed as the light source to ensure consistent lighting conditions.

### 2.2. Device Design of Smartphone Based Blood Parameter Sensing Platform

**Figure 1:**
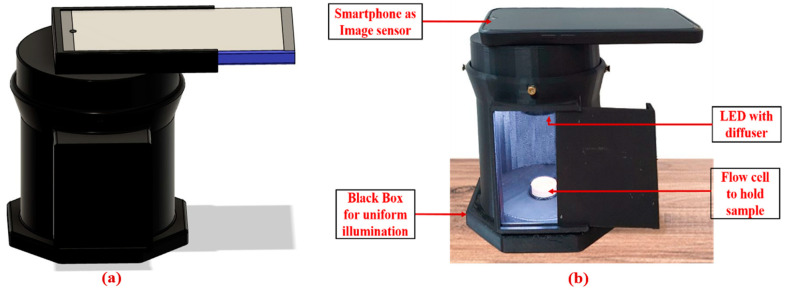
Smartphone-based Blood Parameter Sensing platform; (**a**) 3D CAD model design; (**b**) developed platform.

In this work, a fully integrated, portable, colorimetric smartphone-based blood parameter sensing platform was developed and successfully utilized for various kidney function biomarkers detection, shown in Figure 1. The platform works by analyzing the color change that occurs when a reagent reacts with blood serum, which varies based on the concentration of uric acid in the sample. This color change was then precisely measured using advanced image processing algorithms. For accurate sensing, it is crucial to capture a clear image of the test sample. The 3D-printed enclosure effectively minimizes exposure to external environmental factors, such as fluctuations in ambient temperature and humidity, by creating a controlled and stable testing environment that effectively blocks external light interference. The enclosure was fabricated using black-colored PLA filament to maintain the integrity of the captured images. An LED strip was installed on the top surface to provide the necessary illumination, and a white acrylic sheet was used to diffuse the light, ensuring soft and even illumination. To further enhance the diagnostic process, we developed an Android application that captures real-time images of the test sample, accelerating the overall diagnosis. This application was successfully tested and can be used with any Android-based smartphone.

### 2.3. Flow Cell Fabrication

**Figure 2:**
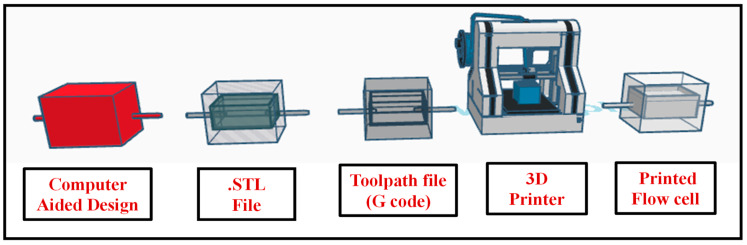
Three-dimensional printing process flow for fabrication of disposable flow cell.

Flow cells play a pivotal role in blood parameter sensing, serving as the essential sensing zone that holds test samples during analysis. In this work, a novel disposable flow cell mechanism was introduced to eliminate the risk of cross-contamination between samples. Fabricated using advanced 3D printing technology with Polylactic Acid (PLA), these flow cells were engineered for exceptional precision and efficiency. The process began with the creation of a detailed computer-aided design (CAD) file that captured the complex geometry of the flow cell. This digital design was then converted into a Standard Tessellation Language (.stl) file, ensuring that every aspect of the flow cell was accurately represented. The final step involved generating Geometric code (G-code), which directed the 3D printer to replicate the CAD model with high fidelity. The fabricated flow cell, having diameter 10 mm and depth 4 mm, holds the sample of 300 µL. This seamless integration of software and hardware resulted in the precise fabrication of the disposable flow cell mechanism, as illustrated in Figure 2. The PLA-based flow cells have demonstrated long-term stability when stored under normal room temperature conditions. Extended testing confirmed that they maintain structural integrity and functionality over time. Rigorous testing was conducted with various reagents used in the system, and the results indicate that these reagents do not cause any degradation or color changes to the flow cells, ensuring consistent performance across multiple tests. Since the developed system does not expose the flow cells to extreme temperatures, their long-term stability is further enhanced under typical operating conditions.

### 2.4. Sample Preparation Protocol

The samples were prepared using the reagents and calibration standard specific to the targeted biomarker. The reagent is responsible for the generation of color after reacting with the analyte of interest. This minimizes cross-reactivity and interference from other biomolecules present in the sample. The calibration standard is the known concentration of targeted analyte required for calibration and sample preparation of other concentrations. For calibration and testing purpose, three types of sample, i.e., Blank, Standard, and Test, were prepared as given in Table 1. For the Blank Sample, 500 µL of reagent was added without any additional concentration of targeted analyte, serving as the control to establish a baseline for colorimetric assessment. The standard sample was prepared by adding 5 µL of a standard solution to 500 µL of the reagent, essential for calibrating the measurement system and validating its accuracy. The Test sample, consisting of 500 µL of reagent and 5 µL of blood serum, is tested. This concentration was determined by comparing the colorimetric deviation against the standard.

A precise volume of the blood serum sample (5 µL) or standard solution is mixed with 500 µL of reagent to ensure uniform and complete interaction between the reagent and analyte. The mixture is incubated at 37 °C for 10 min to ensure that the enzymatic reactions have sufficient time to develop the colorimetric response. This incubation step ensures thermal stability before testing begins and prevents any potential adverse effects of temperature fluctuations during the testing. The flow cell is designed to hold a fixed sample volume, preventing cross-contamination between tests and ensuring precise optical path lengths for accurate absorbance measurements. Using a micropipette, 300 µL of the prepared mixture is carefully introduced into the 3D-printed disposable flow cell to maintain the consistency in the volume of test sample.

The uric acid, creatinine, and albumin reagents include calibration standards of 8 mg/dL, 2 mg/dL, and 4 g/dL, respectively. For the initial standard solution, 5 µL of the standard is added to the 500 µL reagent, ensuring consistency during calibration. To generate test samples with varying concentrations, different volumes of the standard solution are diluted in 500 µL of reagent. The procedure remains the same for uric acid, creatinine, and albumin; however, the specific reagent used and the resulting concentrations differ, as detailed in the Supplementary Material. This approach enables the preparation of multiple concentration levels, essential for calibration curves and accurate quantification of the analytes. The selected calibration range ensures that both low and high concentrations are detected with precision, improving the platform’s diagnostic reliability in diverse scenarios.

### 2.5. Adaptive Calibration Protocol for Device Stability

**Figure 3:**
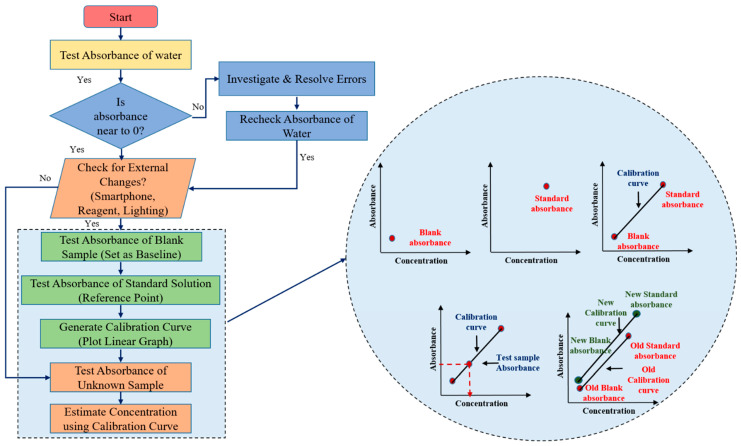
Flowchart of adaptive calibration and estimation of output concentration from calibration curve.

The calibration process for the developed smartphone-based biosensing platform is a critical step to ensure that the device delivers accurate and reliable test results. By establishing a relationship between the absorbance readings and the known concentrations of analytes from standard solutions, the platform can accurately quantify unknown blood parameters. The platform incorporates an adaptive calibration algorithm that recalibrates the factor each time a test begins. This allows it to adjust automatically to any minor variations caused by environmental changes, ensuring accurate and consistent results across varying conditions, such as changes in smartphones, reagents, or environmental factors like lighting, making it highly versatile for real-world applications. The flowchart of adaptive calibration algorithm is as illustrated in Figure 3.

#### 2.5.1. Initial Water Testing

The first step of the calibration process is to test the absorbance of water. Since water contains no targeted analyte, it is expected to show zero absorbance. This step acts as a preliminary check for any potential disturbances or errors in the system. If the absorbance of water deviates from zero, it signals that there may be an issue, such as contamination, sensor malfunction, or interference, and the system needs to be checked for potential sources of error. If the absorbance is zero as expected, the calibration process continues to the next step.

#### 2.5.2. Blank Sample Testing to Establish a Baseline

The absorbance of a blank sample (typically a reagent without the analyte) is then measured. This reading serves as the baseline absorbance for the system, against which all future sample measurements will be compared. This step helps detect any potential background noise or deviations caused by the smartphone’s sensor characteristics. The blank absorbance compensates for any intrinsic absorbance of the reagents or the system that may influence the measurements, ensuring that the detected absorbance is solely due to the presence of the analyte.

#### 2.5.3. Standard Solution Testing to Create a Calibration Curve

Following the blank measurement, the absorbance of a known standard solution is tested. The standard solution contains a precise concentration of the analyte, and its absorbance value provides a reference point for the calibration process. Multiple test solutions with different known concentrations of the biomarker including blank and standard are used during the initial calibration process to generate a comprehensive calibration curve. This curve establishes the relationship between absorbance and concentration across the platform’s detection range. Once the initial multi-point calibration has been established and validated for linearity, the system uses a single standard point as part of the real-time adaptive calibration process. This curve establishes a direct relationship between absorbance and analyte concentration. The slope of this linear curve becomes the foundation for calculating unknown concentrations from the absorbance readings of test samples.

#### 2.5.4. Calculating the Concentration of Unknown Samples

Once the calibration curve is established, the system is ready to test unknown samples. The absorbance of the unknown sample is measured and compared against the calibration curve. Based on the linear relationship, the system calculates the concentration of the analyte in the unknown sample by interpolating its absorbance on the curve.

#### 2.5.5. Ensuring Device Independence and Flexibility

This adaptive calibration process effectively decouples the device’s performance from the specific smartphone model, reagent manufacturer, or environmental setup. As a result, the platform can operate reliably under varying conditions without requiring recalibration for every minor fluctuation in these parameters. The ability to generate a new calibration curve when conditions change makes the biosensing platform more flexible and independent of external variables. This is particularly important for applications where the device might be used in different clinical settings, by different users, or with different supplies, as it allows for real-time adjustments to ensure accuracy. By performing calibration at the beginning of every test session, the platform decouples its performance from the specific smartphone being used. This ensures that the calibration factor remains relevant for the current session and does not rely on previously stored values. As a result, variations in smartphone cameras, lighting conditions, and environmental settings are automatically adjusted.

### 2.6. Data Acquisition and Processing Using Smartphone

**Figure 4:**
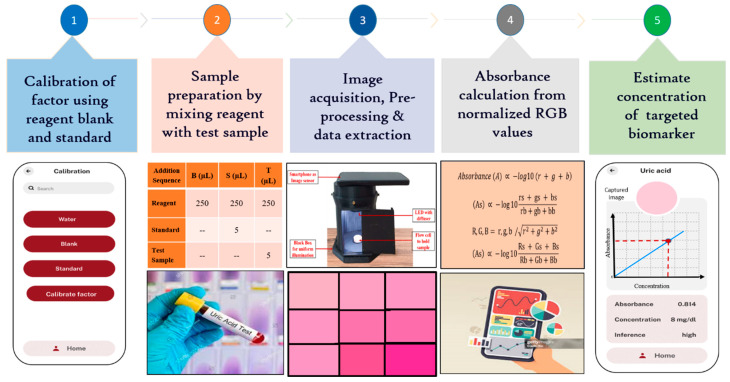
Stepwise algorithm to estimate the uric acid concentration.

The portable smartphone-based biosensing platform for kidney function biomarkers represents a significant advancement in healthcare diagnostics. This platform enables rapid, on-site detection and quantification of critical biomarkers, streamlining the diagnostic process by reducing the need for traditional lab-based testing. With its real-time analysis capability, healthcare providers can make quicker, data-driven decisions, potentially leading to earlier diagnosis and treatment of kidney-related conditions. The platform portability and ease of use empower both clinicians and patients, facilitating continuous monitoring and improving patient outcomes through timely intervention. Figure 4 outlines the workflow of a smartphone-based biosensing platform for biomarker detection.

#### 2.6.1. Image Acquisition and Preprocessing

After sample preparation, it was placed in a precisely 3D-printed flow cell, which was fitted seamlessly into the detection unit beneath the smartphone’s image sensor. A custom-designed Android app was developed to capture the color change induced by the analyte-specific reaction within the flow cell, utilizing the smartphone’s camera. Images captured by different smartphones are standardized by resizing them to a common resolution while preserving the aspect ratio. This ensures that the platform can process images consistently, regardless of the native resolution of the device. Once the image was captured, the image processing techniques, using the OpenCV library, were used to ensure accuracy under varying lighting conditions. White balance correction when implemented, maintains consistent color representation regardless of ambient light variations. Instead of analyzing the entire image, the software focuses on a defined region of interest (ROI) (50 × 50 pixels) where the sample is located. By cropping and analyzing only the relevant portion of the image, the system minimizes the influence of differing resolutions and sensor specifications across devices. Additionally, normalization, performed through reference-based intensity scaling with a blank sample, corrects illumination inconsistencies. The preprocessed image was cropped to a 50 × 50-pixel region of interest (ROI) to focus on the relevant area. The app averaged the cropped images using spatial averaging, which reduced random noise, enhanced the signal-to-noise ratio, and improved both detail and dynamic range. This process resulted in clearer, more consistent images, revealing subtle details that were crucial for precise measurements. The adaptive algorithm then processes the image and extracts normalized RGB values to accurately calculate the concentration of the targeted biomarker while minimizing any noise from non-specific interactions [9].

#### 2.6.2. Relating Light Intensity to RGB Values

RGB values representing the intensity of red, green, and blue light are essential for determining the color and brightness of an image captured by a smartphone. When a chemical solution is photographed, these values reveal key details about how light is absorbed and transmitted through the solution, providing important insights into its color characteristics.

#### 2.6.3. Image Normalization and Absorbance Measurement

To ensure consistent results across different smartphones, lighting conditions, and reagent batches, the platform employs normalization of RGB values based on a reference blank sample. This normalization corrects for variations due to device-specific characteristics, lighting differences, and reagent properties, as explained in detail in the Supplementary Materials. The normalized RGB values are used to calculate the transmitted light intensity (I_t_) and the incident light intensity (I_0_), enabling the estimation of absorbance using the Beer–Lambert law. The mathematical derivations for this process and the calibration of the system are provided in the Supplementary Materials.

### 2.7. Android Application Development

The Android application plays a crucial role in estimating the targeted analyte concentration by streamlining the diagnostic process. Its ability to streamline the image capture and analysis in real-time significantly accelerates diagnosis and treatment. By utilizing the smartphone’s camera to record RGB values of the captured images, the app provides an immediate and accurate measure of targeted analyte concentration, which is essential for efficient and timely decision-making. This real-time analysis not only enhances the speed of the diagnostic process but also supports PoCT applications. The app enables on-site, rapid assessment without the need for complex laboratory equipment, making it ideal for use in various settings, such as clinics, remote areas, or even at home. By integrating this technology, healthcare providers can offer faster, more accessible testing, ultimately improving patient care and response times.

**Figure 5:**
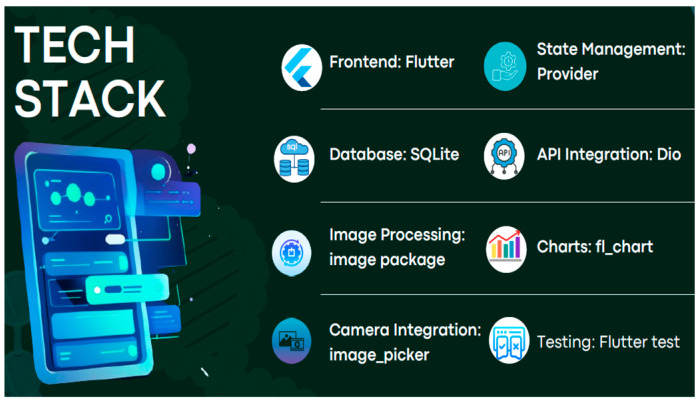
Tech stack used for application development and operation.

The development of the Android application for concentration calculation was a multifaceted process involving various technologies and methodologies, as given in the tech stack in Figure 5. The development process involved several key steps, from designing the frontend interface to integrating image processing and implementing testing procedures. By leveraging Flutter for frontend development, Provider for state management, SQLite for local storage, Dio for API integration, image package for image processing, fl_chart for data visualization, image_picker for camera integration, and Flutter test for testing, a robust and user-friendly application was created. This app allows users to accurately calculate concentrations from image analysis, providing operational flexibility and reliability in diverse conditions.

The frontend of the application was developed using Flutter, a popular framework for building natively compiled applications for mobile from a single codebase. Flutter was chosen for its efficiency in creating smooth and visually appealing UIs. The user interface was designed to be intuitive and user-friendly, allowing users to easily capture images, view results, and manage data. Flutter’s rich set of pre-designed widgets was leveraged to create a cohesive and responsive design. Provider was used for state management to handle the application’s state efficiently. This package allows for a clear separation of UI and design logic, making the application more maintainable and scalable. To store data locally, SQLite was used. This lightweight database solution is well-suited for mobile applications and ensures that users can access their data even without an internet connection.

CRUD (Create, Read, Update, Delete) Database operations were implemented to manage data such as captured images, calculated concentrations, and historical records. Flutter’s sqflite package facilitated the integration with SQLite. For network operations and communication with external services, the Dio package is used. Dio is a powerful HTTP client for Dart, providing features such as request cancelation, interceptors, and response transformation. The application is designed to communicate with potential cloud services or external databases for syncing data, fetching updates, and other network-related tasks. The image package is used for processing images captured by the smartphone camera. This package provides tools for manipulating images, such as resizing, cropping, and color adjustments. The core functionality of the application, which involves extracting RGB values and performing image normalization, is implemented using the image package. This processing is crucial for accurate absorbance and concentration calculations.

**Figure 6:**
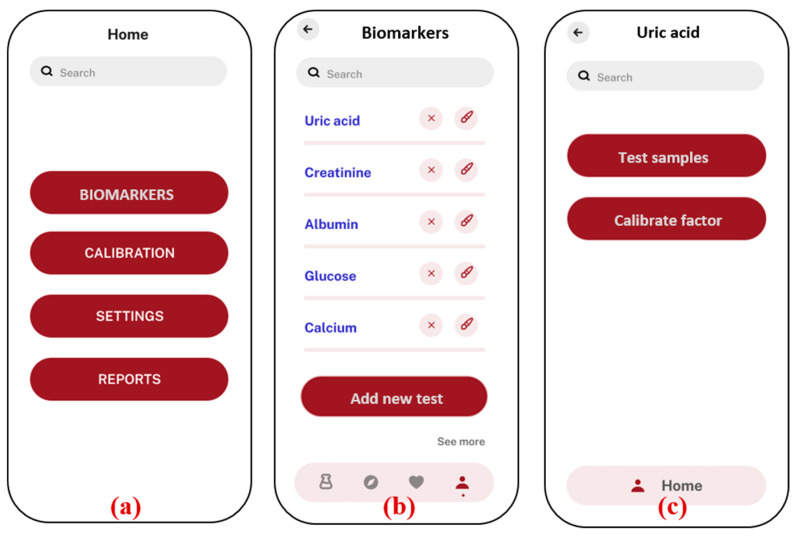

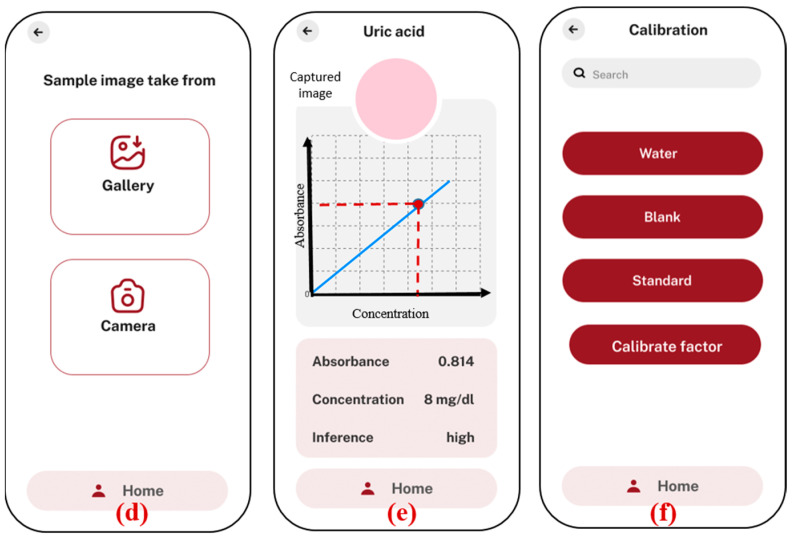
Android application screenshots; (**a**) home screen; (**b**) list of biomarkers with option to add new test; (**c**) uric acid test is selected, option given to test samples with previous factors, and new factors are calibrated; (**d**) option to select the image from the gallery or to take a new photo of sample; (**e**) analysis and results showing absorbance and concentration value, along with the inference with normal ranges in human blood serum; (**f**) Calibration menu to calculate new factors in case of any modifications in external parameters.

To visualize data and display results graphically, the fl_chart package is used. This package supports various types of charts and is highly customizable. The application includes features to display charts representing concentration trends, absorbance values, and other analytical data. These visual aids help users interpret and understand the results better. The image_picker package is used to integrate the smartphone camera into the application. This package allows users to capture images or select existing ones from their gallery. The application provides an interface for users to take pictures of samples and reference images. The image_picker package ensures a seamless and user-friendly experience in capturing and uploading images. To ensure the reliability and performance of the application, extensive testing is conducted using the Flutter test package. This package allows for the creation of unit tests, widget tests, and integration tests. Comprehensive test cases are written to validate the core functionalities of the application, such as image processing, database operations, and UI interactions. Automated testing helped identify and fix bugs early in the development process. The Android application developed for biochemical blood parameter sensing is designed to provide an intuitive and streamlined experience for users involved in biomarker analysis, as shown in Figure 6.

The application starts with a home screen (a), where users can navigate to different sections, such as biomarker selection, calibration, settings, and reports. In the biomarker section (b), users can choose from a list of specific biomarkers, such as uric acid, creatinine, and albumin. The “add new test” option allows users to add new biomarker tests that are based on the colorimetric principle. The new added tests will appear on the same biomarkers screen. Once a biomarker is selected (c), the user is given the option to test the samples directly with the previously stored factors, in case the prior testing conditions are not altered. If there is any change in the smartphones or chemicals, the user needs to recalibrate the factors by using the “calibrate factor” function, which is necessary for accurate analysis. The application offers flexibility in image capture (d), allowing users to either upload an image from the smartphone’s gallery or directly use the camera inside the app. This image is then processed to determine the concentration of the biomarker by analyzing the absorbance values based on the calibration graph (e). The concentration result, along with an inference regarding its level, is displayed on the same screen, ensuring that users can quickly interpret the data. To maintain accuracy, the application includes a calibration section (f), where users can adjust settings based on water, blank, and standard samples. This calibration ensures that the results obtained are precise and reliable. Overall, the application provides a user-friendly interface for conducting biomarker analysis using a smartphone, integrating sophisticated image processing with ease of use.

## 3. Results and Discussion

### 3.1. Sensing of Kidney Biomarker Using Developed Smartphone Based Platform

The developed smartphone-based biosensing platform was used to quantize the concentration of uric acid, creatinine, and albumin in the blood serum required for the monitoring of kidney diseases. For uric acid sensing, the samples were prepared of varying concentrations, ranging from 1 to 30 mg/dL. The methodology used for uric acid sensing was based on its oxidation by the enzyme uricase, as shown in Equations (1) and (2) [21].

Uric Acid + O_2_ + H_2_O → Allantoin + CO_2_ + H_2_O_2_ (1)

DHBS + 4AAP + 2H_2_O_2_ → Quinoneimine + 4H_2_O (2)

**Figure 7:**
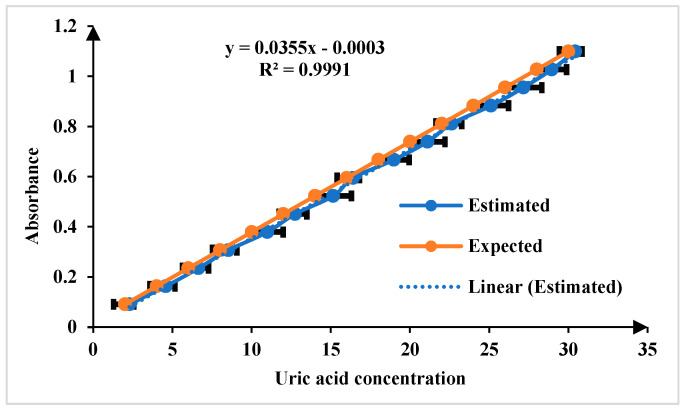
Comparison of expected and estimated uric acid in the linear range of 1 to 30 mg/dL for *n* = 5.

The color change in the Quinoneimine dye serves as an indicator of uric acid concentration, with lighter colors corresponding to lower concentrations and deeper colors to higher concentrations. This color variation is crucial for accurately determining uric acid levels in the sample, which is captured using smartphone camera and analyzed using the app. The absorbance of all the images was analyzed and the concentration of uric acid was estimated. Figure 7 illustrates the calibration curve plotted between uric acid concentration and absorbance, which serves as the foundation for quantifying uric acid levels in this work. The expected and estimated uric acid concentration values demonstrate the accuracy and precision of the developed platform. The linearity of the calibration curve, extending across the tested concentration and ranging from 1 to 30 mg/dL and LoD, is 1.15 mg/dL (*n = 5*, where *n* represents the number of replicate measurements). The LOD was calculated using the formula $LOD=\frac{n\times std.deviation}{slope\;of\;calibrating\;curve}$.

**Figure 8:**
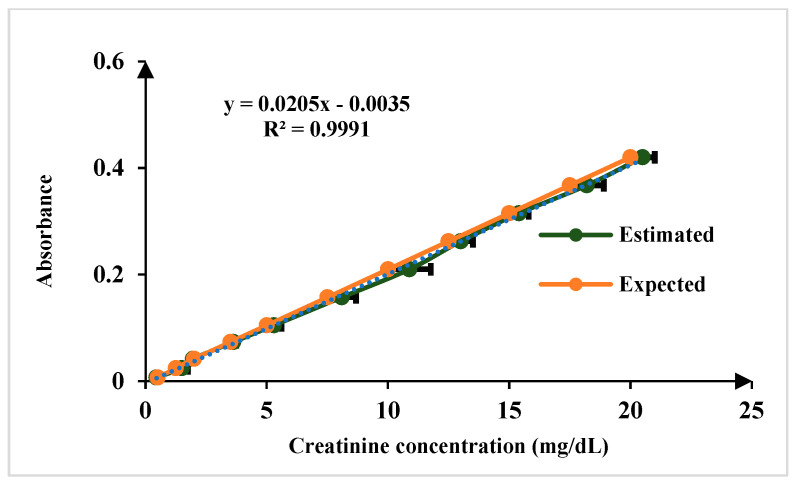
Comparison of results of known concentration of creatinine test samples for *n* = 5.

**Figure 9:**
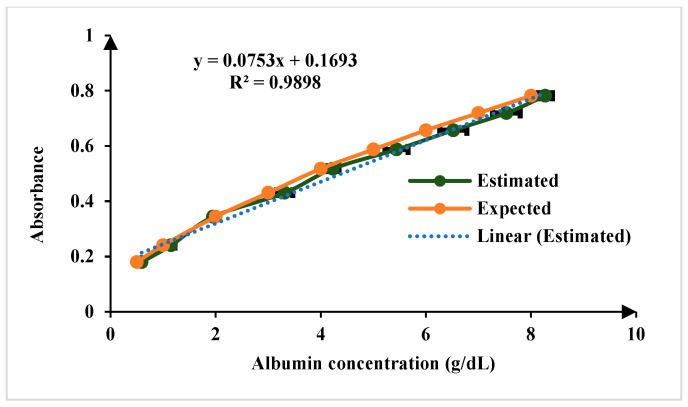
Comparison of results of known concentration of albumin test samples for *n* = 5.

For the quantification of creatinine in blood serum samples, modified Jaffe’s method adopted in this approach. In the Jaffe reaction, creatinine reacts with picric acid in an alkaline environment, forming a complex that exhibits a yellow color. The intensity of the color produced is directly proportional to the concentration of creatinine in the sample [22,23]. The linear range of detection for creatinine sensing is 0.1 to 20 mg/dL, encompassing both normal and elevated concentrations. The normal physiological range of creatinine in human serum is 0.6 to 1.3 mg/dL, ensuring the platform’s capability to distinguish fluctuations within this range while also detecting elevated levels indicative of kidney dysfunction. The limit of detection (LoD) is 0.15 mg/dL for *n* = 5, as illustrated in Figure 8. For sensing albumin in blood serum, the Bromocresol Green (BCG) method is a used in this work. Bromocresol green is a pH-sensitive dye that undergoes a color change when it binds to albumin in acidic conditions. The BCG dye binds to albumin, forming a green complex. The intensity of the green color is then measured using the developed platform, which correlates with the albumin concentration in the sample [23,24]. The linear range of detection for albumin sensing is 0.1 to 8 g/dL, which effectively covers the normal physiological range of 3.7–5.8 g/dL and LoD is 0.11 mg/dL for *n* = 5 as illustrated in Figure 9.

The chosen standard concentrations may not directly match the typical physiological range of the biomarkers but are designed to cover a broader range.

### 3.2. Adaptive Caliberation and Operational Flexibility

The core advantage of the developed adaptive algorithm is its ability to function independently of external parameters, such as the specific smartphone model, the manufacturer of chemicals used, or different external lighting conditions. Different smartphone manufacturers can differ significantly in terms of camera resolution, sensor quality, and image processing capabilities. These differences can affect the RGB values captured in images, leading to potential discrepancies in absorbance calculations. Different chemical manufacturers may have different absorbance properties, which can affect the calibration of the algorithm. Different lighting conditions can also significantly influence the RGB values captured in images. The adaptive algorithm addresses this issue by recalibrating factors each time a new smartphone or chemical is used. By recalibrating, the algorithm ensures that the concentration measurements remain accurate regardless of the external variations. This adaptability is crucial for maintaining consistent measurement accuracy across diverse conditions. By incorporating recalibration steps for each of these variables and offering operational flexibility, the algorithm provides accurate and reliable concentration measurements in a wide range of scenarios. This adaptability ensures that the measurement tool remains robust and effective, regardless of the changes in the device or chemical used as illustrated in Table 2. It displays uric acid concentrations (2 to 20 mg/dL) and shows how these measurements fluctuate when using different devices (Samsung, OnePlus, Motorola) and chemical brands (Erba, Trace, Proton) under various lighting conditions (morning, afternoon, evening). The average variation percentage, calculated for each concentration level, indicates the consistency of the measurements.

### 3.3. Repeatability Analysis

**Figure 10:**
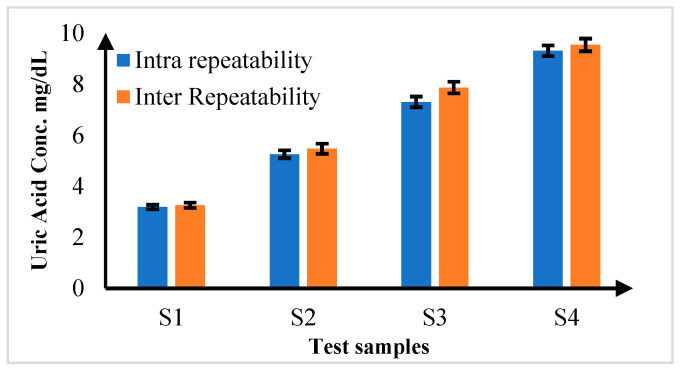
Inter and Intra Repeatability Analysis for uric acid samples for *n* = 5.

In the comprehensive repeatability analysis, the consistency of measurement of the proposed diagnostic platform was assessed by conducting a series of tests under varied conditions. First, intra-repeatability was evaluated by performing five repeated measurements (*n* = 5) on the same sample under identical conditions. The results showed exceptionally low percentage coefficients of variation (%CV), ranging from 0.039% to 0.103%. These values indicate remarkable precision, demonstrating that a prosed platform consistently delivers accurate results when the same sample is tested multiple times under the same conditions. Inter-repeatability was analyzed by measuring different samples of the same concentration under varying conditions. These experiments were conducted with five repeated measurements (*n* = 5) for each sample at different concentration levels. The %CV values for inter-repeatability ranged from 0.198% to 0.516%. While these values were slightly higher than those for intra-repeatability, they still reflect robust precision, indicating that the platform reliably measures samples of the same concentration despite changes in testing conditions. The detailed results are summarized in Figure 10, which highlights the high level of precision and consistency achieved by the proposed colorimetric system.

### 3.4. Validation of the Developed Colorimetric Platform

To validate the performance of the developed biochemical blood-sensing platform, the results were compared with those obtained from a commercial Trace40 biochemistry analyzer. The comparison covered several parameters, including uric acid, creatinine, and albumin. The platform’s design, including sample incubation and adaptive calibration, ensures that minor variations or potential interactions do not affect the accuracy of detection. Validation tests, as given in Table 3, have shown that the platform consistently correlates well with commercial analyzers, demonstrating its robustness even in complex biological samples. The concentration ranges were chosen to cover both normal and abnormal values commonly observed in clinical settings, ensuring the platform’s practical utility for detecting early abnormalities and monitoring disease progression. Additionally, the platform’s ability to detect incremental colorimetric shifts allows for the testing of other biomarkers, potentially extending its use to monitor diseases related to the heart, liver, and pancreas. This flexibility underscores the platform’s potential for broad diagnostic applications in clinical settings.

The accuracy of the developed biosensing platform was further validated using a spike-and-recovery approach for uric acid measurements. Known concentrations of uric acid were spiked into test samples, and the recovered concentrations were compared against the expected values. The recovery percentage was calculated using the following formula: $Recovery\left(\%\right)=\left(\frac{Measured\;concentration\left(Spiked\;sample\right)-Baseline\;concentration}{Expected\;Spike\;Concentration}\right)\times 100$. The results, presented in Table 4, demonstrate recovery rates ranging from 95.5% to 98.5%, indicating minimal matrix interference and high analytical accuracy. These findings confirm the platform’s capability to reliably quantify uric acid in complex biological samples, reinforcing its suitability for real-world diagnostic applications.

## 4. Conclusions

The developed smartphone-based biosensing platform for kidney biomarker sensing presents a portable, accurate, and user-friendly alternative to traditional diagnostic devices. The smartphone-based platform is inherently more cost-effective because it leverages existing smartphone hardware, significantly reducing the need for expensive, dedicated instrumentation. The 3D-printed flow cells used in this platform are low-cost and easily replaceable, further enhancing the cost-effectiveness while maintaining clinical precision. The developed platform provides greater flexibility through its adaptive calibration algorithm, which allows it to dynamically adjust to variations in environmental conditions, such as lighting, smartphone models, or reagent differences. The validation of the platform against commercial analyzers, alongside repeatability tests, has confirmed its accuracy and reliability, with minimal variation across different test conditions.

With an intuitive app-based interface, the platform ensures ease of use, requiring minimal training for operation in clinical and field settings. While it is highly effective for biomarkers producing measurable color changes, complex biological samples with overlapping signals may need further optimization. Overall, this technology improves accessibility to diagnostic testing, particularly in low-resource settings, by combining affordability, reliability, and practicality for point-of-care applications. Future advancements, including AI-based image analysis, multi-wavelength sensing, cloud integration, automated sample handling, and enhanced optical components, will further expand its diagnostic capabilities.

## Figures and Tables

**Table 1:** Preparation of test samples for Uric Acid, Creatinine, and Albumin.

Preparation Protocol	Blank	Standard	Test Sample
Reagent	500 µL	500 µL	500 µL
Standard	--	5 µL	--
Test Sample	--	--	5 µL
Inference	No concentration of targeted analyte, used for baseline	Known concentration of targeted analyte, used for calibration	Unknown concentration of targeted analyte, to be determined

**Table 2:** Average concentration variation (%) of uric acid test sample across different smartphones, chemical brands, and lighting conditions (*n* = 5).

Actual Uric Acid Concentration mg/dL	Different Smartphone, Chemical Brand, and Lighting Conditions (*n* = 5)			Average Variation %
	Samsung	One Plus	Motorola	
	Erba	Trace	Proton	
	Morning	Afternoon	Evening	
2	2.16	2.05	2.19	6.67
4	4.17	4.28	3.8	5.42
8	8.27	7.31	7.41	6.46
12	11.03	11.14	11.85	5.50
20	20.85	20.41	20.81	3.45

**Table 3:** Result Validation with Commercial Analyzer.

Test	Analyzer	Results				
Uric acid (mg/dL)	Commercial	4.15	8.23	2.01	5.25	1.24
	Developed	4.72	8.49	2.29	5.88	1.51
Creatinine (mg/dL)	Commercial	1.23	1.41	2.52	3.18	3.56
	Developed	1.20	1.37	2.48	3.11	3.41
Albumin (g/dL)	Commercial	5.2	8	2	4.2	6.1
	Developed	5	7.3	1.8	4	5.9

**Table 4:** Spike-and-Recovery Analysis for Uric Acid Using the Developed Analyzer.

Sample	Baseline Concentration (mg/dL)	Spiked Concentration (mg/dL)	Measured Concentration (mg/dL)	Recovery (%)
S1	2.01	2.00	3.95	97.5
S2	4.15	4.00	8.05	98.0
S3	5.25	3.00	8.12	95.8
S4	8.23	2.50	10.64	96.4
S5	1.24	1.50	2.72	98.5

## Data Availability

The original contributions presented in the study are included in the article; further inquiries can be directed to the corresponding authors.

## Supplementary Materials

The following supporting information can be downloaded at: https://www.mdpi.com/article/10.3390/bios15030192/s1, Table S1. Preparation of test samples with varying concentrations for Uric Acid, Creatinine, and Albumin.
