# Supplementary material for: A Portable Smartphone-Based 3D-Printed Biosensing Platform for Kidney Function Biomarker Quantification
Source: Biosensors (Basel). 2025 Mar 18;15(3):192. doi: 10.3390/bios15030192 (PMC11939896; doi:10.3390/bios15030192)
Supplement: Supplementary file 1 [file biosensors-15-00192-s001.zip › biosensors-3406233-supplementary.pdf]

Supplementary Material

Estimation of uric acid concentration from image captured using smartphone. The captured image undergoes following pre and post processing steps:

1. Normalization of RGB values

Normalization is critical as it accounts for variations in color intensity and ensures that the analysis is not skewed by inconsistent lighting or other external factors. Normalization typically involves scaling these values to a specific range, such as [0, 1], which enhances the performance and reliability of image processing algorithms. This standardization makes color values comparable across different images and conditions, improving the precision of the analysis. The standard deviation (SD) of RGB components is integral to this process as it measures the variability of color intensity values from their mean. By scaling RGB values relative to their standard deviation, the normalization process adjusts for overall color intensity, ensuring that the analysis is both accurate and sensitive to variations in RGB values. To normalize RGB values, first calculate the standard deviation using the below formula

$$\mathrm{SD}=\sqrt{R^2 + G^2 + B^2} \qquad (1)$$

Then, normalize each RGB component by dividing it by the standard deviation.

$$Rnorm = \frac{R}{\sqrt{R^2+G^2+ B^2}},\ Gnorm = \frac{G}{\sqrt{R^2+G^2+ B^2}},\ Bnorm = \frac{B}{\sqrt{R^2+G^2+ B^2}} \qquad (2)$$

Normalization of RGB values is crucial for minimizing variability caused by differences in smartphone camera sensors, lighting conditions, and reagent sources. Without normalization, raw RGB values can be inconsistent due to device-specific characteristics, such as white balance and exposure, or slight changes in external lighting. To address these issues, the platform normalizes RGB values using the standard deviation of a blank sample’s RGB components. This approach has been shown to improve both stability and precision in colorimetric biosensing systems (Priye et al., 2018; Roy et al., 2018). Normalization accounts for differences in resolution, white balance, and exposure settings among different smartphones, allowing consistent absorbance calculations across devices (Xu et al., 2018). Even under controlled lighting, ambient variations can affect image capture. By scaling RGB values based on the blank sample’s standard deviation, the platform reduces these effects, ensuring reproducible results (Roy et al., 2018). The scaling of RGB values reduces noise and allows the system to detect subtle colorimetric changes in the sample, critical for accurate concentration estimation. The normalization procedure mitigates formulation differences between reagent sources, ensuring reliable performance across diverse reagent brands. This normalization method is particularly suited for point-

of-care applications, where conditions can vary significantly, making reliable image-based measurements essential for accurate diagnostics.

2. Calculate light intensity

In the context of RGB values from digital images, light intensity represents the brightness of the light detected by each of the red, green, and blue sensors in the imaging device. Once the RGB values are normalized, the transmitted light intensity I_t can be estimated. The transmitted light intensity is a measure of the average light intensity across the red, green, and blue channels. The composite transmitted intensity value is calculated by averaging the normalized RGB values obtained from the captured image of test sample.

$$I = \frac{R_{t,norm} + G_{t,norm} + B_{t,norm}}{3} \qquad (3)$$

The incident light I_0 is the intensity of light before it passes through a sample. The incident intensity is calculated using the normalized RGB values of a blank sample image that reflects or transmits the light without significant absorption. This reference provides a baseline measurement of the light intensity before it interacts with the sample. Extract the red R_0, green G_0, and blue B_0 values from the blank sample image. Compute the standard deviation of the RGB values, which represents the overall intensity of the light in the reference image.

$$SD_0 = \sqrt{R_0^2 + G_0^2 + B_0^2} \qquad (4)$$

Normalize each RGB component of the reference image by dividing it with SD_0. Since the reference is expected to reflect or transmit the incident light without significant absorption, the normalized RGB values ideally sum up to represent the total incident light intensity:

$$I_0 = \frac{R_{0,norm} + G_{0,norm} + B_{0,norm}}{3} \qquad (5)$$

3. Calculation of absorbance

Absorbance is a measure of how much light is absorbed by a substance as it passes through a sample. In this case, the absorbance of light is dependent on the uric acid molecules present in the test sample. Absorbance (A) measures the reduction in light intensity as light passes through a sample. The Beer-Lambert Law provides a quantitative relationship between absorbance and concentration. It is defined by the logarithmic ratio of the incident light intensity to the transmitted light intensity:

$$Absorbance\ (A) = \log_{10}\left(\frac{I_0}{I_t}\right) \qquad (6)$$

Where the incident light intensity (I_0) is the intensity of light before it enters the sample. Transmitted Light Intensity (I_t) is the intensity of light after it passes through the sample.

$$A = Log_{10}(\frac{R_{0,norm} + G_{0,norm} + B_{0,norm}}{R_{t,norm} + G_{t,norm} + B_{t,norm}}) \quad (7)$$

4. Estimation of targeted analyte concentration

In the linear range of the Beer-Lambert Law, absorbance is directly proportional to the concentration of the substance.

$$A = \varepsilon * C * l \quad (8)$$

Where, A is the absorbance, ε is the molar absorptivity, C is the concentration, l is the path length.

Hence,

$$C = \frac{A}{\varepsilon * l} \quad (9)$$

To calculate the concentration of uric acid, initially the absorbance of standard A_{std} is calculated,

$$C_{std} = \frac{A_{std}}{\varepsilon_{std} * l_{std}} \quad (10)$$

Then, the absorbance of test sample whose uric acid concentration is to be estimated is calculated,

$$C_t = \frac{A_t}{\varepsilon_t * l_t} \quad (11)$$

The length of light travel and molar absorptivity is assumed to be constant for constant medium and by taking ratios,

$$\frac{C_{std}}{C_t} = \frac{A_{std}}{A_t} \quad (12)$$

Finally, the concentration of uric acid in the test sample is calculated as follows,

$$C_t = \frac{A_{std}}{C_{std}} * A_t \quad (13)$$

$$C_t = Factor * A_t \quad (14)$$

The ratio $\frac{A_{std}}{C_{std}}$ is termed as factor and output concentration can be obtained by multiplying this factor with the absorbance of the sample to be tested. For any analyte sensing, the standard concentration is known and the absorbance of standard is calculated from equation (7). Now, the only unknown variable in the equation (14) is the absorbance of the unknown test sample. Using this the concentration of targeted analyte in any unknown blood serum sample can be estimated.

Sample Peparation protocol

The procedure of sample preparation remains the same for uric acid, creatinine, and albumin; however, the specific reagent used and the resulting concentrations differ, as detailed in Table S1. The chosen standard concentrations may not directly match the typical physiological range of the biomarkers but are designed to cover a broader range. Real-world samples may exhibit biomarker

concentrations outside the normal range due to varying clinical conditions, such as kidney disease progression or acute cases. The selected calibration range ensures that both low and high concentrations are detected with precision, improving the platform's diagnostic reliability in diverse scenarios.

Table S1. Preparation of test samples with varying concentrations for Uric Acid, Creatinine, and Albumin.

**Uric acid**															
**Test sample to prepared of concentration mg/dL**	2	4	6	8	10	12	14	16	18	20	22	24	26	28	30
**Concentration of standard to be added in 500 μL of reagent in μL**	1.25	2.5	3.75	5	6.25	7.5	8.75	10	11.3	12.5	13.8	15	16.3	17.5	18.8

**Albumin**															
**Test sample to prepared of concentration g/dL**	1	1.5	2.0	2.5	3.0	3.5	4.0	4.5	5.0	5.5	6.0	6.5	7.0	7.5	8.0
**Concentration of standard to be added in 500 μL of reagent in μL**	1.3	1.9	2.5	3.1	3.8	4.4	5	5.6	6.3	6.9	7.5	8.1	8.8	9.4	10

**Creatinine**															
**Test sample to prepared of concentration mg/dL**	0.5	1	2	3.5	5	6.5	8	9.5	11	12.5	14	15.5	17	18.5	20
**Concentration of standard to be added in 500 μL of reagent in μL**	1.25	2.5	5	8.75	12.5	16.3	20	23.8	27.5	31.3	35	38.8	42.5	46.3	50
